# Fibular hemimelia

**DOI:** 10.25122/jml-2021-0397

**Published:** 2022-04

**Authors:** Tiberiu Georgescu, Olivia Ionescu, Oana Daniela Toader, Nicolae Bacalbasa, Lucian Gheorghe Pop

**Affiliations:** 1. National Institute of Mother and Child Care Alessandrescu-Rusescu, Bucharest, Romania; 2. Department of Obstetrics and Gynecology, Carol Davila University of Medicine and Pharmacy, Bucharest, Romania; 3. Department of Obstetrics and Gynecology, South Nurnberg Hospital, Nurnberg, Germany

**Keywords:** bowed tibia, absent fibula, prenatal diagnosis

## Abstract

Fibular hemimelia is defined as a partial or complete absence of the fibula. Alongside fibular deformities, there is a wide spectrum of anomalies, foot deformities, and absent rays. A literature review showed only a handful of cases of prenatal diagnosis of fibular hemimelia. It is a rare disorder that might be isolated or associated with visceral anomalies.

## Introduction

**Figure 1.**
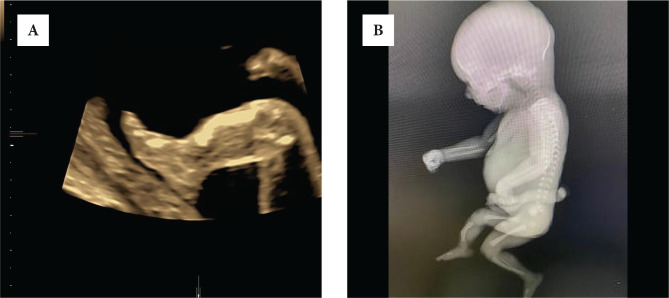

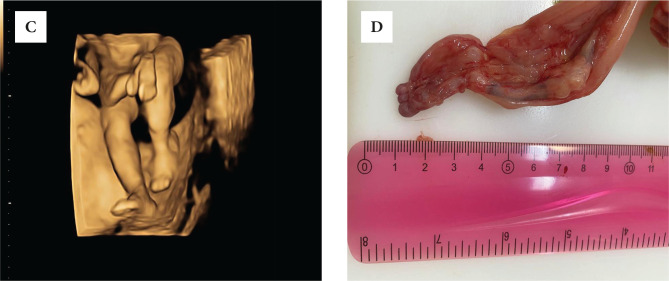
A – Multiplanar 3D bowed tibia and absent fibula; B – X-ray bowed tibia and absent fibula of right leg; C – 3D rendering short leg and three fingers; D – post-aborted specimen bowed tibia and absent fibula and three digits.

We present the case of a 29-year-old woman, 19 weeks pregnant, who underwent a routine second-trimester scan. Her first-trimester scan was unremarkable. Following her scan, fetal abnormalities were noted on the left foot. There was an absent fibula with a short and bowed tibia. On further examination, the absence of the fourth and fifth toes was observed (Figure 1 A–D). The rest of the bones were within normal limits. The scan was performed using a convex probe 2D-4D RAB 6 D Voluson E10 BT16 (General Electric Healthcare, Ultrasound Zipfer, Austria). Except for the fibula and toes, the fetal skeletal system was symmetrically and appropriately developed, as were the hands. No other organ anomalies were identified. Because of the findings mentioned above, a diagnosis of fibular hemimelia, most likely type 2, was made. Following detailed counseling, the parents decided to end the pregnancy. Nevertheless, umbilical cord blood was obtained at delivery, and SNP microarray analysis showed a normal result (Agilent Cytogenomics 3.0.2.11 software). The post-mortem exam confirmed the antenatal findings.

## Discussion

In embryological life, the lower extremities are sonographically seen before the upper extremities, whereas movement of upper extremities is seen before the movement of lower extremities. The long fetal bones can be measured from 11 weeks onward [1]. Fibular hemimelia is an extremely rare disorder, with inconsistency regarding its actual incidence. Complete fibular aplasia, mild fibular aplasia, and unilateral or bilateral fibular hemimelia are all part of the same family. The unilateral form is more common than the bilateral form at a ratio of 3 to 1 [2]. Typically, in unilateral cases, anterior bowing of the tibia occurs, and the right side is more commonly involved. In almost all cases, there is a toe deficiency. The etiology of fibular hemimelia is unclear, but several theories, such as an absent anterior tibial artery, can lead to defects in muscle development and flaws in the apical ectodermal ridge [3]. Another theory is a disruption of the limb during embryogenesis. The preferred classification is the one published by Achterman and Kalamachi, which relies on clinical and radiological features. Class I with minimal hypoplasia of the fibula and Class II with fibula aplasia. Our case is type II fibular hemimelia [4]. Postnatally there are different surgical treatments, but when there is a significant discrepancy, amputation with early use of prosthesis is recommended [5]. 

## Conclusion

Our case shows a type II fibular hemimelia with fibular aplasia, tibial bowing, and toes deficiency. Our sonographic diagnosis was confirmed post-abortum. The parents chose to terminate the pregnancy at 19 weeks gestation. An accurate diagnosis is possible, and new scanning technologies can improve our knowledge enabling us to provide better counseling for parents, which should be done in a multidisciplinary format, encompassing obstetricians, neonatologists, geneticists, and pediatrician surgeons.

## Acknowledgments

### Conflict of interest

The authors declare no conflict of interest.

### Consent to participate

Informed consent to publish the data was obtained from the participant.

### Authorship

TG performed the autopsy. OI and ODT wrote the original draft and edited the pictures. NB reviewed the article. LGP performed the scan and managed the case.
